# Radiation Therapy for Retroperitoneal Sarcomas: Influences of Histology, Grade, and Size

**DOI:** 10.1155/2018/7972389

**Published:** 2018-12-05

**Authors:** Jennifer L. Leiting, John R. Bergquist, Matthew C. Hernandez, Kenneth W. Merrell, Andrew L. Folpe, Steven I. Robinson, David M. Nagorney, Mark J. Truty, Travis E. Grotz

**Affiliations:** ^1^Department of Surgery, Mayo Clinic, Rochester, MN 55901, USA; ^2^Department of Radiation Oncology, Mayo Clinic, Rochester, MN 55901, USA; ^3^Department of Pathology, Mayo Clinic, Rochester, MN 55901, USA; ^4^Department of Medical Oncology, Mayo Clinic, Rochester, MN 55901, USA

## Abstract

Perioperative radiation therapy (RT) has been associated with reduced local recurrence in patients with retroperitoneal sarcomas (RPS); however, selection criteria remain unclear. We hypothesized that perioperative RT would improve survival in patients with RPS and would be associated with pathological factors. The National Cancer Database (NCDB) from 2004 to 2012 was reviewed for patients with nonmetastatic RPS undergoing curative intent resection. Tumor size was dichotomized at 15 cm based on 8th edition American Joint Committee on Cancer (AJCC) staging. Patients with the highest comorbidity score were excluded. Unadjusted Kaplan–Meier and adjusted Cox proportional hazards modeling analyzed overall survival (OS). Multivariable logistic regression modeled margin positivity. A total of 2,264 patients were included; 727 patients (32.1%) had perioperative radiation in whom 203 (9.0%) had radiation preoperatively. Median (IQR) RPS size was 17.5 [11.0–27.0] cm. Histopathology was high grade in 1048 patients (43.7%). Multivariable analysis revealed that perioperative radiation was independently associated with decreased mortality (HR 0.72, 95% confidence intervals (CIs) 0.62–0.84, *p* < 0.001), and preoperative RT was associated with reduced margin positivity (HR 0.72, 95% CI 0.53–0.97, *p*=0.032). Stratified survival analysis showed that radiation was associated with prolonged median OS for RPS that were high-grade (64.3 vs. 43.6 months, *p* < 0.001), less than 15 cm (104.1 vs. 84.2 months, *p*=0.007), and leiomyosarcomatous (104.8 vs. 61.8 months, *p* < 0.001). Perioperative radiation is independently associated with decreased mortality in patients with high-grade, less than 15 cm, and leiomyosarcomatous tumors. Preoperative radiation is independently associated with margin-negative resection. These data support the selective use of perioperative radiation in the multidisciplinary management of RPS.

## 1. Introduction

Retroperitoneal sarcomas (RPS) are a subset of soft tissue sarcomas (STS) that provide an oncologic treatment challenge. An R0 surgical resection provides patients with the best chance for improved survival and decreased local recurrence [1, 2], but R0 resection often is difficult to achieve because of large tumor size and frequent involvement of adjacent structures and organs [3]. Indeed, the anatomy of the retroperitoneum makes it difficult, if not impossible, to obtain negative margins compared to extremity STS. Consequently, local recurrence is the most common pattern of disease progression after surgical resection of RPS [4, 5]. Furthermore, three out of every four deaths due to RPS are the result of local recurrence. Therefore, better local control is needed to improve survival [6].

Adjuvant radiation therapy (RT) has been associated with reduced local recurrence in a randomized trial for extremity sarcomas [7], but no such trial has been completed for RPS. Although multiple prior database studies have shown RT was associated with improved survival with extremity sarcoma, only patients with high-grade sarcomas benefited, regardless of location [8–11]. Among prior database studies for RPS, there have been mixed results with some showing a survival benefit with adjuvant radiation and others showing no improvement [12–14]. The influence of tumor size, grade, and histology on the outcomes associated with perioperative RT for patients with RPS remains unclear and has not previously been studied. Although the National Comprehensive Cancer Network (NCCN) recommends preoperative RT in all RPS [15], in practice, the decision to treat patients with perioperative RT remains controversial and inconsistent across the United States (US), given the lack of available evidence [16].

Further data are needed to improve decision-making regarding selection of patients most likely to benefit from RT. Tumor size, grade, and histological subtype have been shown to be the determinants of survival after resection of RPS [17, 18]. We sought to determine whether these factors were associated with survival in patients with RPS who were treated with perioperative RT. We hypothesized that perioperative RT would be associated with an overall improvement in outcomes, but also that tumor size, grade, and histologic subtype would influence these outcomes. To test this hypothesis, we used the National Cancer Database (NCDB) to determine survival outcomes in a large cohort of patients treated for RPS.

## 2. Materials and Methods

This retrospective cohort analysis of the NCDB participant user file (PUF) examined patients undergoing treatment for RPS from 2004–2012. The Institutional Review Board at Mayo Clinic has deemed analysis of the NCDB PUF exempt. The NCDB contains over 30 million records of individual cancer cases in a national hospital-based registry collected by more than 1,500 Commission on Cancer- (CoC-) approved facilities across the US. The NCDB data reporting is tracked and audited, and must meet quality standards in order for centers to maintain their CoC center designation [19]. The NCDB has previously been shown to capture approximately 70% of all new cases of cancer in the US [20].

**Figure 1:**
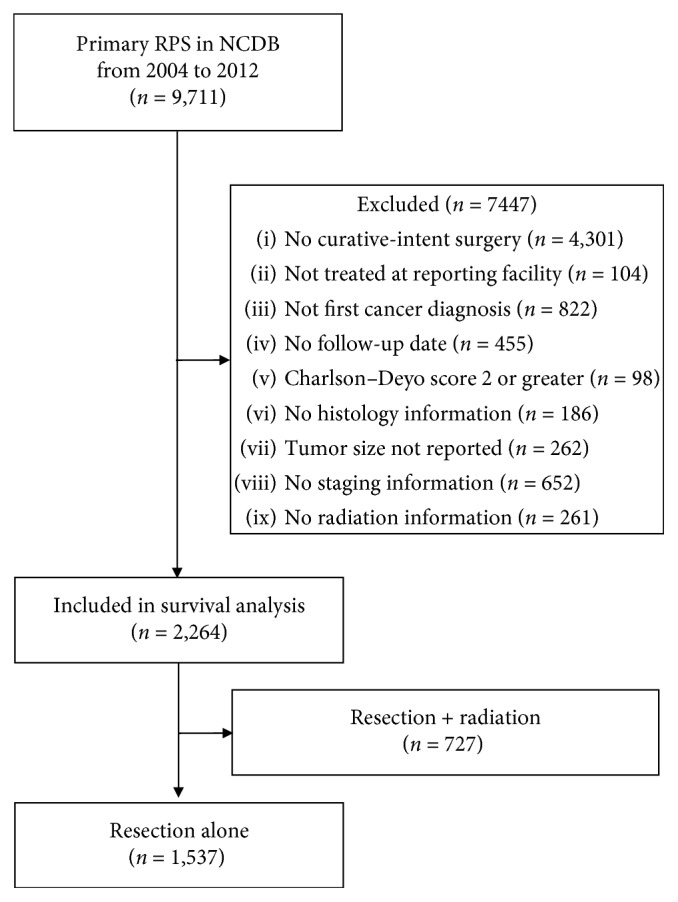
STROBE diagram of patients included in the study.

Patients with RPS were identified using International Classification of Diseases for Oncology, 3rd edition (ICD-O-3) topography (C48.0) and histology (8800–8806, 8810–8815, 8830, 8840, 8850–8858, 8890–8896, 8900–8902, 8910–8912, and 8980–8982) codes. Only patients diagnosed and treated at the reporting facility were included. Patients diagnosed with cancer at more than one site, treated as part of a palliative plan of care, or missing data on staging or follow-up were excluded. Comorbidity was assessed using the method outlined by Deyo et al. [21], and patients with a comorbidity score of 2 or greater were excluded due to their increased risk of early noncancer-related mortality. Only patients treated with curative-intent surgery were included (surgery of primary site codes 30–60). A STrengthening the Reporting of OBservational studies in Epidemiology (STROBE) compliant diagram showing the patients included and excluded in the study is shown in Figure 1.

### 2.1. Statistical Analysis

Unadjusted Kaplan–Meier survival analysis was performed with survival defined from date of diagnosis, and survival estimates were compared with the log-rank test. Probability of survival at interval time horizons is reported with 95% confidence intervals (CIs). A 0.05 level of significance was used for all comparisons. All statistical tests were two-tailed. Statistical analysis was performed with R version 3.2.4 [22].

## 3. Results

A total of 9,711 patients were identified with primary RPS. Of these, 7,447 patients (76.7%) were excluded for reasons listed in Figure 1, and 2,264 patients (23.3%) were included in the final analysis. Of these patients, 1,537 (67.9%) were treated with resection alone, 727 patients (32.1%) were treated with resection and perioperative radiation, and 203 patients (8.9%) underwent preoperative radiation. Overall comorbidity and demographic background were similar between treatment groups, except that patients treated with both resection and radiation were younger (Table 1).

For all patients, the median (IQR) tumor size was 17.5 (11.0–27.0) cm and 996 (44.0%) patients had high-grade tumors. Histological subtypes included liposarcoma, leiomyosarcoma, undifferentiated pleomorphic sarcoma, and sarcoma not otherwise specified (NOS). There were 941 (75.8%) well-differentiated liposarcomas and 300 (24.2%) dedifferentiated liposarcomas. Tumor histopathology was significantly different between treatment groups. Patients treated with resection alone were more likely to have liposarcomas than leiomyosarcomas (Table 1). The patients treated with surgery alone also had significantly larger tumors with a median tumor size of 19.0 (12.0–29.0) cm compared to 15.0 (9.0–20.6) cm in the group that received radiation (*p* < 0.001). Not all patients had data on regional radiation dose recorded (664/727, 91.3%); but among those who had dose information available in the record, median radiation dose was 50 Gy (IQR 45–51 Gy). Boost radiation was only given to 147 (20.0%) patients, and they received a median of 10.8 Gy boost radiation (IQR 8.7–16.1 Gy). Patients treated with radiation more frequently had high-grade RPS (51.9% vs 40.3%, *p* < 0.001).

**Figure 2:**
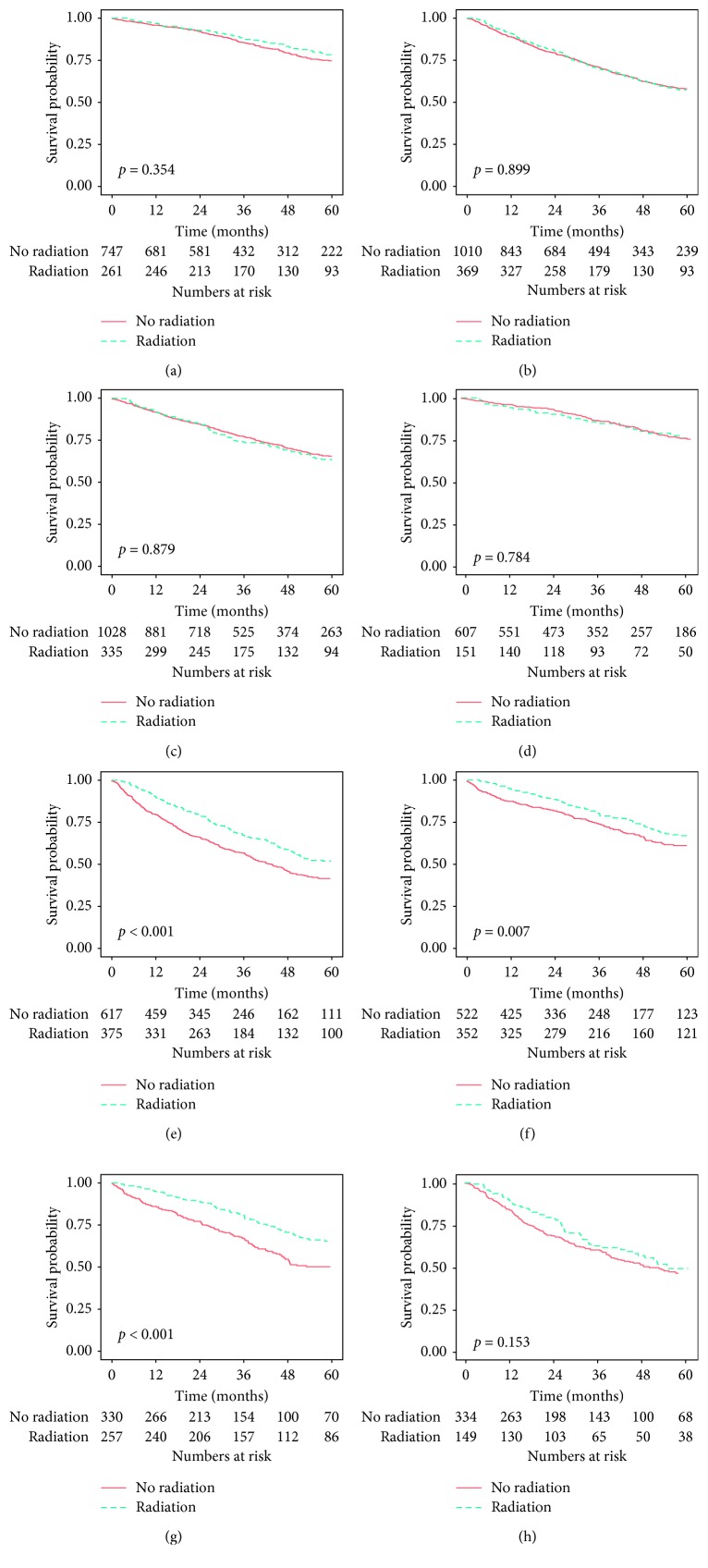
Unadjusted stratified Kaplan–Meier analysis. Low-grade tumors (a), tumors ≥15 cm (b), liposarcoma (c), well-differentiated liposarcoma (d), high-grade tumors (e), tumors <15 cm (f), leiomyosarcoma (g), and dedifferentiated liposarcoma (h).

To assess the effect of radiation on OS in patients with RPS, a survival analysis was performed comparing patients treated with resection alone vs. patients treated with resection and perioperative radiation (Figure 2) stratified by various factors. Three factors significantly correlated with survival: RPS grade, size, and histologic subtype. Median OS was greater for patients after resection and radiation compared to patients treated with resection alone with high-grade RPS (64.3 vs. 43.6 months, *p* < 0.001) and with tumors less than 15 cm (104.1 months vs. 84.2 months, *p*=0.007). Lastly, median OS for patients with leiomyosarcomas was greater after resection and radiation than after resection alone (104.8 months vs. 61.8 months, *p* < 0.001). Overall median survival did not differ between treatment groups for low-grade RPS (*p*=0.354), liposarcomas (*p*=0.879), and RPS ≥15 cm (*p*=0.899). In a subgroup analysis of liposarcomas, there was no statistically significant impact on OS for patients undergoing radiation with either well-differentiated or dedifferentiated liposarcomas.

**Figure 3:**
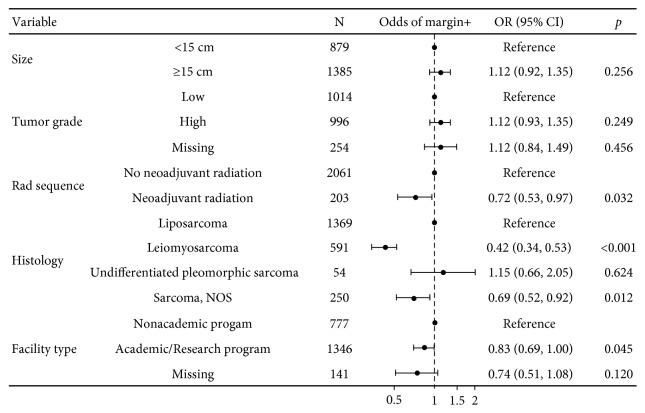
Adjusted model of margin status.

**Figure 4:**
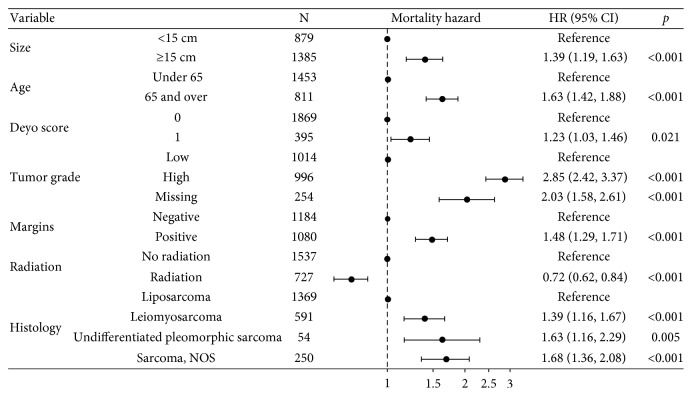
Adjusted model of overall survival.

An adjusted multivariable analysis of resection margin status showed that neoadjuvant radiation was associated with a reduction of positive margins (OR 0.72, 95% CI 0.53–0.97, *p*=0.032) (Figure 3). The RPS size and grade did not correlate with margin status. A multivariable analysis of mortality hazard showed that radiation was independently associated with a decreased mortality hazard (HR 0.72, 95% CI 0.62–0.84, *p* < 0.001) (Figure 4). Factors that were independently associated with increased mortality hazard were tumors ≥15 cm, age greater than 65, high-grade RPS, positive margins, and RPS histological subtypes including leiomyosarcoma, undifferentiated pleomorphic sarcoma, and sarcoma NOS.

## 4. Discussion

This study of the NCDB, a large national registry representing more than 70% of newly diagnosed malignancies nationwide, suggests that perioperative radiation as a surgical adjunct to improve local control may translate selectively into an improvement in OS. The survival benefit of radiation persisted after adjustment for other factors that influence survival including age, comorbidity, margin status, tumor size, grade, and histologic subtype. This finding supports the current NCCN guidelines for consideration of perioperative RT for all RPS. However, our study clarifies those guidelines by identifying those patients with RPS most likely to benefit from radiation: small RPS, high-grade RPS, and leiomyosarcomas. Although a randomized clinical trial addressing radiation and resection would best confirm these findings, large registry studies will likely provide the best further validation of the benefit of radiation on OS for RPS. Additional data assessing local control by radiation for patients undergoing resection for RPS is needed. Because three out of every four deaths due to RPS are caused by local recurrence, the impact of radiation on local control is likely a key factor affecting survival. However, the clinical impact of perioperative radiation on OS related to different subsets of RPS and relationships to adjacent organs and structures may further affect treatment selection.

The role of neoadjuvant radiation for patients with RPS is particularly relevant, given its potential impact on achieving R0 resection. The ability of preoperative radiation to improve margin status for RPS has been reported in our study, as well as in a previous retrospective study [23]. This effect on margins has been seen in pancreas cancer as well and is thought to be associated with the direct treatment effect of the radiation [24]. A randomized controlled trial of preoperative radiotherapy 50.4 Gy/28 fractions followed by en-bloc resection of RPS compared to en-bloc resection of RPS alone (Surgery With or Without Radiation Therapy in Untreated Nonmetastatic Retroperitoneal Sarcoma (STRASS) trial NCT01344018) has been undertaken by the European Organisation for Research and Treatment of Cancer (EORTC) with the primary endpoint of abdominal recurrence-free survival. Results are anticipated soon and are expected to validate the improvement in local recurrence-free survival reported in small nonrandomized studies. However, the trial will not be powered for the secondary end-point of OS.

The actual impact of radiation with resection has been difficult to assess because of the incidence and heterogeneity of RPS and inconsistent selection of patients in its application. Although the NCCN guidelines recommend perioperative radiation for all RPS [15], we found fewer than one-third of patients received perioperative radiation nationally and less than 28% of these patients received the radiation preoperatively. Identification of clinical and pathological factors which predict patients with the greatest benefit from the addition of perioperative radiation may help to improve national adherence to treatment guidelines. We identified high-grade tumors, the histological subtype of leiomyosarcoma, and tumors less than 15 cm to have the most dramatic benefit in OS with the addition of perioperative radiation. Our data are consistent with previous reports illustrating that the radioresponsiveness of STS is variable and dependent on grade and histology [25]. Higher histologic grade has been associated with higher local recurrence rates and decreased survival [5]. Radiation therapy has been postulated to help mitigate these risks for high-grade tumors and is further supported in our study [26]. Histologic differentiation and grade are important determinants of clinical outcomes in liposarcoma patients. Well-differentiated retroperitoneal liposarcomas mostly present as local recurrences but do not metastasize, with a five-year OS of 90% [5]. In contrast, dedifferentiated and pleomorphic liposarcomas have poor outcomes compared to well-differentiated but still preferentially recur in a local pattern. Given this distinction, it was surprising to find no significant difference in OS for perioperative radiation in well-differentiated and dedifferentiated liposarcoma groups on subgroup analysis. Yet, it is important to remember that this study was unable to look at other important clinical outcomes, like recurrence-free survival, which play a significant role in the disease process of liposarcomas.

Although no large series exists on the radioresponsiveness of leiomyosarcoma, there is a general agreement on the necessity and benefit of perioperative RT. A previous report of 14 patients with retroperitoneal leiomyosarcoma suggested a significant improvement in local recurrence and OS for those treated with RT in addition to surgery [27]. Similarly, our study suggests a significant improvement in survival for patients with leiomyosarcoma treated with radiation plus resection compared to resection alone. The influence of size likely reflects the challenges of achieving therapeutic radiation levels to a larger treatment field while simultaneously minimizing toxicity. This is particularly challenging when the majority of the radiation in this study was given postoperatively. We postulate that advanced techniques, experience, and preoperative delivery of RT may overcome the limitation of size.

A small subset of patients received chemotherapy as part of their treatment regimen, and it is difficult to determine whether this was administered as part of chemoradiation in the context of the NCDB. Chemotherapy is not a standard treatment for RPS [28], and chemoradiation is rarely used outside of clinical trials. For these reasons, chemotherapy status was not included in the survival analysis as its impact is likely insignificant in this cohort.

Our study is limited by its retrospective and nonrandomized nature which we have attempted to address through the use of multivariable modeling to adjust for known confounders. There is no information on patterns and time of recurrence in the NCDB. Survival analysis thus is limited to OS. We attempted to account for this by excluding patients who had significant comorbidity scores and thus competing risks of death. We have utilized outlier exclusion and multivariable regression instead of propensity matching in order to maximize the study's power to detect differences in the populations under study. We have previously discussed the limitations of propensity matching for retrospective cohort analysis, and do not feel they would add much to this study [29, 30]. Indications for radiation and its delivery are unknown.

## 5. Conclusion

Despite the growing evidence to support improved OS through local control by the addition of radiation to resection of RPS, only a minority of patients who present with curative-intent resection for RPS will be given any perioperative radiation, and even fewer patients will receive preoperative radiation. Our investigation of the NCDB suggests that radiation is independently associated with decreased mortality. This benefit is particularly evident in patients with high-grade, less than 15 cm, and leiomyosarcomatous tumors. As a result, perioperative radiation for RPS should be selectively considered in the multidisciplinary management of RPS.

## Figures and Tables

**Table 1:** Patient clinical and demographic characteristics categorized by receipt of perioperative radiation.

	Resection alone	Resection + radiation therapy	*p*
	*n*=1,537	*n*=727	
Median age (IQR)	61 [52–70]	59 [51–68]	0.002
Female sex	53.0%	53.2%	0.939
Race			0.034
Caucasian	84.4%	80.9%	
African American	9.7%	13.3%	
Others	5.9%	5.8%	
Charlson-Deyo score			0.607
0	82.2%	83.2%	
1	17.8%	16.8%	
Facility type			<0.001
Nonacademic	31.5%	40.3%	
Academic/research	62.9%	52.1%	
Missing	5.6%	7.6%	
Histology			<0.001
Liposarcoma	67.1%	46.4%	
Leiomyosarcoma	21.5%	35.8%	
Undifferentiated Pleomorphic			
Sarcoma	2.0%	3.2%	
Sarcoma, NOS	9.3%	14.7%	
Operation			0.514
Simple/partial resection	28.5%	26.1%	
Total resection	23.9%	25.7%	
Debulking	4.9%	5.8%	
Radical resection	42.6%	42.4%	
Chemotherapy			<0.001
No chemotherapy	86.7%	79.8%	
Chemotherapy	10.7%	16.9%	
Missing	2.7%	3.3%	
Median tumor size (IQR) (cm)	19.0 [12.0–29.0]	15.0 [9.0–20.6]	<0.001
Tumor size category			<0.001
<15 cm	36.8%	52.5%	
>15 cm	63.2%	47.5%	
Grade			<0.001
Low	48.8%	36.3%	
High	40.3%	51.9%	
Missing	10.9%	11.8%	
Margin status			<0.001
Negative	52.6%	51.7%	
Positive	32.1%	39.6%	
Missing	15.3%	8.7%	
30-day readmission (unplanned)	6.1%	5.0%	<0.001
Median hospital stay (days)	6	6	0.177
90-day mortality	4.3%	2.1%	0.013
Median OS (months)	82.7	95.8	<0.001
Interval survival (95% CI)			
1 year	88.3%	92.7%	
2 year	80.1%	84.2%	
3 year	71.7%	74.9%	
5 year	58.9%	61.6%	
10 year	NR	4.0%	

## Data Availability

The deidentified participant user file used to support the findings of this study was supplied by the NCDB under license and cannot be made freely available. Requests for access to this data should be made to NCDB at ncdb@facs.org.

## References

[1] Anaya D. A., Lev D. C., Pollock R. E. (2008). The role of surgical margin status in retroperitoneal sarcoma. *Journal of Surgical Oncology*.

[2] Lewis J. J., Leung D., Woodruff J. M., Brennan M. F. (1998). Retroperitoneal soft-tissue sarcoma: analysis of 500 patients treated and followed at a single institution. *Annals of Surgery*.

[3] Raut C. P., Pisters P. W. T. (2006). Retroperitoneal sarcomas: combined-modality treatment approaches. *Journal of Surgical Oncology*.

[4] Bonvalot S., Rivoire M., Castaing M. (2009). Primary retroperitoneal sarcomas: a multivariate analysis of surgical factors associated with local control. *Journal of Clinical Oncology*.

[5] Singer S., Antonescu C. R., Riedel E., Brennan M. F. (2003). Histologic subtype and margin of resection predict pattern of recurrence and survival for retroperitoneal liposarcoma. *Annals of Surgery*.

[6] Stojadinovic A., Yeh A., Brennan M. F. (2002). Completely resected recurrent soft tissue sarcoma: primary anatomic site governs outcomes. *Journal of the American College of Surgeons*.

[7] Yang J. C., Chang A. E., Baker A. R. (1998). Randomized prospective study of the benefit of adjuvant radiation therapy in the treatment of soft tissue sarcomas of the extremity. *Journal of Clinical Oncology*.

[8] Koshy M., Rich S. E., Mohiuddin M. M. (2010). Improved survival with radiation therapy in high-grade soft tissue sarcomas of the extremities: a SEER analysis. *International Journal of Radiation Oncology, Biology, Physics*.

[9] Schreiber D., Rineer J., Katsoulakis E. (2012). Impact of postoperative radiation on survival for high-grade soft tissue sarcoma of the extremities after limb sparing radical resection. *American Journal of Clinical Oncology*.

[10] Lazarides A. L., Eward W. C., Speicher P. J. (2015). The use of radiation therapy in well-differentiated soft tissue sarcoma of the extremities: an NCDB review. *Sarcoma*.

[11] Ramey S. J., Yechieli R., Zhao W. (2018). Limb-sparing surgery plus radiotherapy results in superior survival: an analysis of patients with high-grade, extremity soft-tissue sarcoma from the NCDB and SEER. *Cancer Medicine*.

[12] Choi A. H., Barnholtz-Sloan J. S., Kim J. A. (2012). Effect of radiation therapy on survival in surgically resected retroperitoneal sarcoma: a propensity score-adjusted SEER analysis. *Annals of Oncology*.

[13] Tseng W. H., Martinez S. R., Do L. (2011). Lack of survival benefit following adjuvant radiation in patients with retroperitoneal sarcoma: a SEER analysis. *Journal of Surgical Research*.

[14] Giuliano K., Nagarajan N., Canner J. K. (2016). Predictors of improved survival for patients with retroperitoneal sarcoma. *Surgery*.

[15] von Mehren M., Randall R. L., Benjamin R. S. (2014). Soft tissue sarcoma, version 2.2014. *Journal of the National Comprehensive Cancer Network*.

[16] Porter G. A., Baxter N. N., Pisters P. W. T. (2006). Retroperitoneal Sarcoma: a population-based analysis of epidemiology, surgery, and radiotherapy. *Cancer*.

[17] Raut C. P., Miceli R., Strauss D. C. (2016). External validation of a multi-institutional retroperitoneal sarcoma nomogram. *Cancer*.

[18] Tan M. C., Brennan M. F., Kuk D. (2016). Histology-based classification predicts pattern of recurrence and improves risk stratification in primary retroperitoneal sarcoma. *Annals of Surgery*.

[19] Bilimoria K. Y., Stewart A. K., Winchester D. P., Ko C. Y. (2008). The National Cancer Data Base: a powerful initiative to improve cancer care in the United States. *Annals of Surgical Oncology*.

[20] Raval M. V., Bilimoria K. Y., Stewart A. K., Bentrem D. J., Ko C. Y. (2009). Using the NCDB for cancer care improvement: an introduction to available quality assessment tools. *Journal of Surgical Oncology*.

[21] Deyo R. A., Cherkin D. C., Ciol M. A. (1992). Adapting a clinical comorbidity index for use with ICD-9-CM administrative databases. *Journal of Clinical Epidemiology*.

[22] The R Foundation (October 2017). *The R Project for Statistical Computing*.

[23] Nussbaum D. P., Speicher P. J., Gulack B. C. (2015). Long-term oncologic outcomes after neoadjuvant radiation therapy for retroperitoneal sarcomas. *Annals of Surgery*.

[24] Colbert L. E., Hall W. A., Nickleach D. (2014). Chemoradiation therapy sequencing for resected pancreatic adenocarcinoma in the National Cancer Data Base. *Cancer*.

[25] Willett C. G., Schiller A. L., Suit H. D., Mankin H. J., Rosenberg A. (1987). The histologic response of soft tissue sarcoma to radiation therapy. *Cancer*.

[26] Mussi C., Collini P., Miceli R. (2008). The prognostic impact of dedifferentiation in retroperitoneal liposarcoma: a series of surgically treated patients at a single institution. *Cancer*.

[27] Hines O. J., Nelson S., Quinones-Baldrich W. J., Eilber F. R. (1999). Leiomyosarcoma of the inferior vena cava: prognosis and comparison with leiomyosarcoma of other anatomic sites. *Cancer*.

[28] ESMO/European Sarcoma Network Working Group (2014). Soft tissue and visceral sarcomas: ESMO Clinical Practice Guidelines for diagnosis, treatment and follow-up. *Annals of Oncology*.

[29] Bergquist J. R., Thiels C. A., Storlie C. B., Nagorney D. M., Truty M. J. (2016). How matching may impact interpretation: comments on “A matched-cohort analysis of 192 pancreatic anaplastic carcinomas and 960 pancreatic adenocarcinomas: a 13-year North American experience using the National Cancer Data Base (NCDB)”. *Surgery*.

[30] Bergquist J. R., Shubert C. R., Storlie C. B., Habermann E. B., Truty M. J. (2017). Patient selection for neoadjuvant therapy in early-stage pancreatic cancer. *Journal of Clinical Oncology*.

